# SUV39H1 Reduction Is Implicated in Abnormal Inflammation in COPD

**DOI:** 10.1038/srep46667

**Published:** 2017-04-20

**Authors:** Tzu-Tao Chen, Sheng-Ming Wu, Shu-Chuan Ho, Hsiao-Chi Chuang, Chien-Ying Liu, Yao-Fei Chan, Lu-Wei Kuo, Po-Hao Feng, Wen-Te Liu, Kuan-Yuan Chen, Ta-Chih Hsiao, Jer-Nan Juang, Kang-Yun Lee

**Affiliations:** 1 Division of Pulmonary Medicine, Department of Internal Medicine, Shuang Ho Hospital, Taipei Medical University, New Taipei City, Taiwan; 2 Graduate Institute of Clinical Medicine, College of Medicine, Taipei Medical University, Taipei, Taiwan; 3 School of Respiratory Therapy, College of Medicine, Taipei Medical University, Taipei, Taiwan; 4 Division of Pulmonary Medicine, Department of Internal Medicine, School of Medicine, College of Medicine, Taipei Medical University, Taipei, Taiwan; 5 Division of Pulmonary Oncology and Interventional Bronchoscopy, Department of Thoracic Medicine, Linkou Chang Gung Memorial Hospital and Chang Gung University, Taoyuan, Taiwan; 6 Department of Engineering Science, National Cheng Kung University, Tainan, Taiwan; 7 Graduate Institute of Environmental Engineering, National Central University, Taoyuan, Taiwan

## Abstract

Chronic obstructive pulmonary disease(COPD) is characterized by enhanced chronic inflammation in the airways, lung parenchyma, and circulation. We investigated whether SUV39H1, a histone methyltransferase, is causatively implicated in the abnormal inflammation observed in COPD. The SUV39H1 and H3K9me3 levels were reduced in peripheral blood mononuclear cells(PBMCs), primary human small airway epithelial cells(HSAEpCs) and lung tissues from COPD patients, which were correlated with poor lung function and the serum IL-8 and IL-6 levels. A specific SUV39H1 inhibitor, chaetocin, induced a distinct COPD panel of inflammatory cytokines in normal PBMCs. Mechanistically, chaetocin reduced the SUV39H1 and H3K9me3 levels in the native *IL-8* promoter in normal HSAEpCs, which mimicked unstimulated COPD HSAEpCs and led to decreased HP-1α levels and increased RNA polymerase II levels. SUV39H1 knockdown reproduced the pattern of COPD inflammation, whereas SUV39H1 overexpression in COPD HSAEpCs rescued the H3K9me3 levels and suppressed inflammation. In COPD mice, chaetocin further repressed the SUV39H1/H3K9me3 levels and enhanced inflammation. SUV39H1 epigenetically controls a distinct panel of pro-inflammatory cytokines. Its reduction in COPD leads to a loss of the repressive chromatin mark H3K9me3 and confers an abnormal inflammatory response to stimulators. SUV39H1 and its regulatory pathways are potential therapeutic targets for COPD.

Chronic obstructive airway disease(COPD) is a leading cause of morbidity and mortality worldwide^1^. Although the current medications improve symptoms and quality of life, none of them decreases mortality in prospective trials^2^,^3^. Effective treatment will rely on a complete understanding of the disease pathogenesis. COPD, a chronic inflammatory pulmonary disease, is characterized by a progressive airflow limitation that is not fully reversible^4^. An abnormal inflammatory response of the lung to noxious particles or gases, mostly to smoking, appears to play a central role in the disease pathogenesis, in which neutrophils, alveolar macrophages and T lymphocytes are all implicated^4^. Abnormal inflammation has the characteristics of amplification, perpetuation and insensitivity to steroids. Therefore, quitting smoking does not appear to resolve the chronic inflammation^5^. The inflammation is relatively steroid insensitive and can lead to a lack of clinical efficacy of corticosteroids in COPD^6^. The development of novel agents to overcome glucocorticoid insensitivity or to target other critical molecules involved in the inflammation is thus urgently needed.

COPD not only focuses on the lung, but it also has systemic manifestations, such as cardiovascular disease, lung cancer and other malignancies^7^. These comorbidities increase the risk of hospitalization and mortality, which are major causes of death^8^. Systemic inflammation is increasingly recognized as a manifestation of COPD and has been implicated in the systemic effects and excess mortality risk of this disease^9^,^10^. In the ECLIPSE cohort, by quantifying the white blood cell count and the CRP, IL-6, IL-8, fibrinogen and TNF-α levels, a novel phenotype of persistent COPD systemic inflammation was identified^11^. This phenotype is associated with poor clinical outcomes. It remains unclear how this systemic inflammation occurs and is sustained.

The acetylation of specific lysine residues in the core histones, which is controlled by a balance between histone acetylases and histone deacetylase(HDACs), is normally associated with gene activation^12^. In the lungs of COPD patients, reductions in HDAC activity and HDAC2 expression are associated with increases in the *IL-8* mRNA levels and histone-H4 acetylation at the promoter^13^. These activity and expression effects also contribute to glucocorticoid insensitivity^14^. Moreover, the decreased expression of the anti-aging HDAC protein, SIRT1, in the lungs of COPD patients might also be implicated in chronic inflammation^15^. Compared to the extensively studied HDACs in COPD, histone methylation is less understood.

The suppressor of variegation 3–9 homolog 1, SUV39H1, is the prototype SET-domain-containing histone methyltransferase, which specifically catalyzes trimethylation on histone H3K9(H3K9me3) and governs its global level^16^,^17^. H3K9me3 was originally known to play an important role in heterochromatin formation^18^, and it was later implicated in fundamental cellular processes^18^,^19^,^20^,^21^. H3K9me3 mediates gene silencing by recruiting repressors and cofactors, including HDACs and heterochromatin protein-1α(HP1α)^19^. This repressive chromatin mark has been identified as a promoter of a few LPS-inducible inflammatory genes in monocytes and dendritic cells^20^,^21^. The SUV39H1–H3K9me3–HP1α pathway also participates in silencing TH1 loci, thereby ensuring TH2 lineage stability^22^. Interestingly, the dysregulation of SUV39H1-H3K9me3 has been reported as a major underlying mechanism for metabolic memory and the sustained pro-inflammatory phenotype of diabetic cell^23^.

We recently reported that peripheral blood mononuclear cells(PBMCs) from patients with COPD produced more pro-inflammatory cytokines, particularly IL-8, compared to normal subjects and identified reduced NF-κB repressing factor(NKRF) is linked to this systemic inflammation^24^. However, compared with the more complicated inflammation in COPD, NKRF only controls a few genes. In this study, we hypothesized that the dysregulation of SUV39H1 was involved in the abnormal inflammation of COPD. We tested this hypothesis by examining the expression levels in PBMCs and bronchiolar epithelial cells and confirmed its implication by using *in vitro* studies. These results were also confirmed by immunohistochemistry(IHC) analysis of the lung tissues and animal studies.

## Results

### Decreased expression of SUV39H1 in PBMCs from patients with COPD correlated is correlated with systemic inflammation

**Figure 1:**
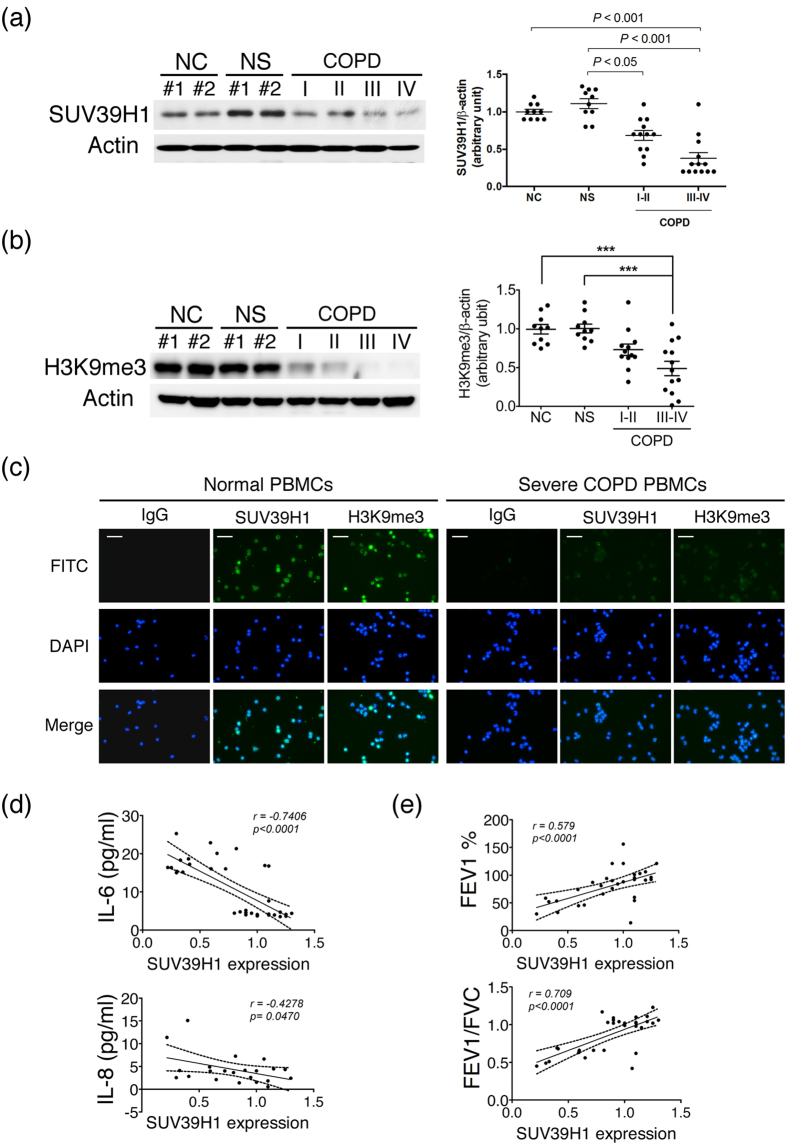
SUV39H1 and H3K9me3 levels are reduced in peripheral blood mononuclear cells(PBMCs) from chronic obstructive pulmonary disease(COPD) subjects. The protein expression in PBMC samples from normal control subjects(NC), normal smoking subjects(NS), and COPD patients were measured by immunoblot.(**a**) The SUV39H1 expression levels were significantly reduced in COPD PBMCs compared with normal or smoking subjects. Actin served as a loading control. Densitometry values for SUV39H1 were normalized to actin. Values were expressed as the fold change over control.(**b**) The global H3K9me3 levels were also diminished in COPD PBMCs.(**c**) Cytospin preparations from freshly isolated PBMCs from normal or severe COPD subjects stained for SUV39H1 and H3K9me3(green color). Cell nuclei were visualized using DAPI(blue color). Isotype controls remained negative, original magnification ×200. All data are presented as the mean ± SEM of at least three independent experiments.(**d**) A significant correlation was observed between the IL-6 and SUV39H1 levels of serum and PBMC samples in all subjects(upper panel). IL-8 levels are associated with SUV39H1 expression in normal non-smoking controls and COPD patients(lower panel).(**e**) Correlation between pulmonary function parameters FEV1 or FEV1/FVC and SUV39H1 in all subjects. The undetermined values of IL-8 are excluded from the analysis. Scale bar, 200 μm. *p < 0.05; **p < 0.01; and ***p < 0.001.

PBMCs were collected from normal healthy controls(NC), normal smoking subjects(NS), and patients with mild to severe COPD. The characteristics of the COPD subjects are summarized in the Methods. To establish the clinical implication of SUV39H1 in COPD, we first analyzed the expression levels of SUV39H1 in PBMCs by western blotting(Fig. 1a). The expression level of SUV39H1 was significantly reduced in PBMCs from patients with COPD compared with those from normal non-smoking(NC) or smoking(NS) controls. Notably, its expression was markedly reduced in patients with more severe COPD(stages III and IV). A similar reduction in the H3K9me3 levels was also observed(Fig. 1b). The reduced expression of both SUV39H1 and H3K9me3 was further confirmed by fluorescence microscopy(Fig. 1c). The positive correlation between the SUV39H1 and H3K9me3 levels in COPD PBMCs is shown in Supplementary Fig. S1A. To investigate whether this phenomenon was associated with the prominent inflammatory cytokine levels in the serum of all subjects, Pearson’s correlation analysis was performed. The IL-6 levels were negatively associated with SUV39H1 expression(Fig. 1e, upper panel). However, the IL-8 levels displayed a trend, although it was not significant, of a negative correlation with SUV39H1 in all subjects(Supplementary Fig. S1B). A previous report showed that IL-8, but not IL-6, appears to be a marker in normal smoking subjects^11^, which suggests that smoking induces systemic IL-8 inflammation. After excluding healthy smokers, we found a significant negative correlation between IL-8 and SUV39H1 expression(Fig. 1e, lower panel). Moreover, there was significant correlation between the lung function index FEV1% predicted, or FEV1/FVC, and SUV39H1 in all subjects(Fig. 1f).

### SUV39H1 inhibition mimics the pattern of COPD inflammation in PBMCs

**Figure 2:**
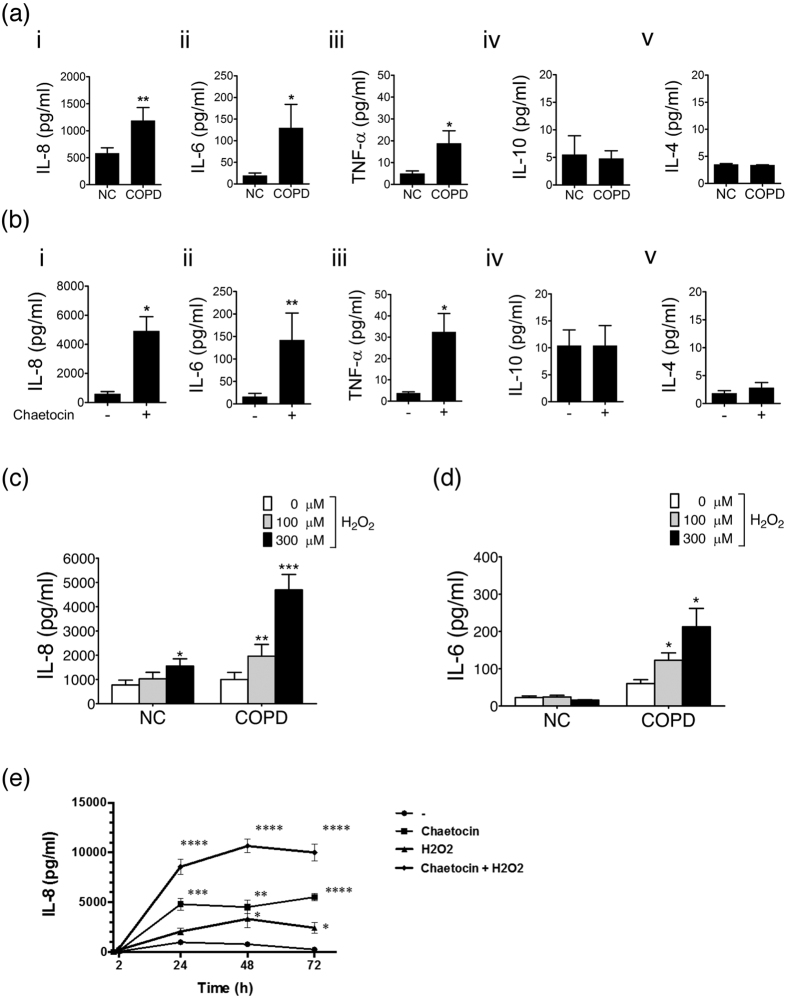
SUV39H1 inhibition induces a pro-inflammatory response in normal PBMCs and a prolonged duration of oxidative stress. (**a**) COPD PBMCs increased pro-inflammatory cytokines, including IL-8, IL-6, and TNF-α, but not IL-10 and IL-4, compared with normal control PBMCs(NC).(**b**) PBMCs were seeded in the presence of SUV39H1 inhibitor, chaetocin, at 100 nM. IL-8 expression in supernatant was measured by ELISA before overnight stimulation. Chaetocin induced a distinct panel of cytokines, which mimicked un-stimulated COPD PBMCs.(**c**) Normal PBMCs were treated with 100 or 300 μM H_2_O_2_ or growth medium as a control for 24 hours, and IL-8 secretion was assessed with an ELISA. IL-8 was significantly induced in a dose-dependent manner in COPD PBMCs with 100 or 300 μM H_2_O_2_.(**d**) The IL-6 level was not induced in normal PBMCs with the same oxidative stress action. However, IL-6 was significantly increased in COPD PBMCs with H_2_O_2_ treatment.(**e**) Chaetocin synergistically enhanced IL-8 production with H_2_O_2_ at 100 μM in a prolonged duration. However, it weakly and maximally stimulated IL-8 production in normal PBMCs with H_2_O_2_ at 48 h compared with both chaetocin and H_2_O_2_ treatment. All data are presented as the mean ± SEM of at least three independent experiments. *p < 0.05; **p < 0.01; ***p < 0.001; and ****p < 0.0001.

COPD PBMCs produce several pro-inflammation cytokines, including IL-8, IL-6, and TNF-α, but not IL-10 or IL-4, compared with normal PBMCs(Fig. 2a). As it has been reported that dysregulation of SUV39H1-H3K9me3 may cause a pro-inflammatory phenotype in diabetic cell^23^, we further investigated whether the down-regulation of SUV39H1 induced inflammatory gene activation in PBMCs. Indeed, the specific SUV39H1 inhibitor chaetocin augmented a distinct panel of pro-inflammatory cytokines that were secreted by normal PBMCs, which did not include Th2 cytokines IL-4 and IL-10(Fig. 2b). Interestingly, this pattern of cytokine production was almost identical to that of COPD PBMCs(Fig. 2a). Similarly, SUV39H1 inhibitor sustained and augmented pro-inflammatory cytokines in COPD PBMC cells(Supplementary Fig. S2A). Therefore, the pharmacological inhibition of SUV39H1 appears to mimic the pattern of COPD inflammation in PBMCs, which supports a pathological role of SUV39H1 reduction in COPD inflammation.

### SUV39H1 inhibition enhances the inflammatory response with a prolonged duration

The effect of pro-inflammation stimulation was assessed in normal or COPD PBMCs. The IL-8 level was slightly increased in normal PBMCs upon H_2_O_2_ stimulation. Significant induction was only seen at 300 μM(Fig. 2c). However, IL-8 was robustly and concentration-dependently increased in COPD PBMCs with 100 or 300 μM H_2_O_2_ stimulation(Fig. 2c). The IL-6 level was unaffected after H_2_O_2_ stimulation in normal PBMCs(Fig. 2d). Similarly, IL-6 was significantly increased in COPD PMBCs with oxidative stress(Fig. 2d). Next, the effect of inflammation stimulation was evaluated in normal PBMCs treated with the SUV39H1 inhibitor chaetocin. Chaetocin alone robustly increased the IL-8 response and synergistically enhanced H_2_O_2_-stimulated production(Fig. 2e). Notably, chaetocin sustained the effect for at least 72 h. These results suggest that the inhibition of SUV39H1 could mimic the augmentation and perpetuation characteristics of inflammation in COPD.

### Down-regulation of SUV39H1 in the lungs of COPD patients

**Figure 3:**
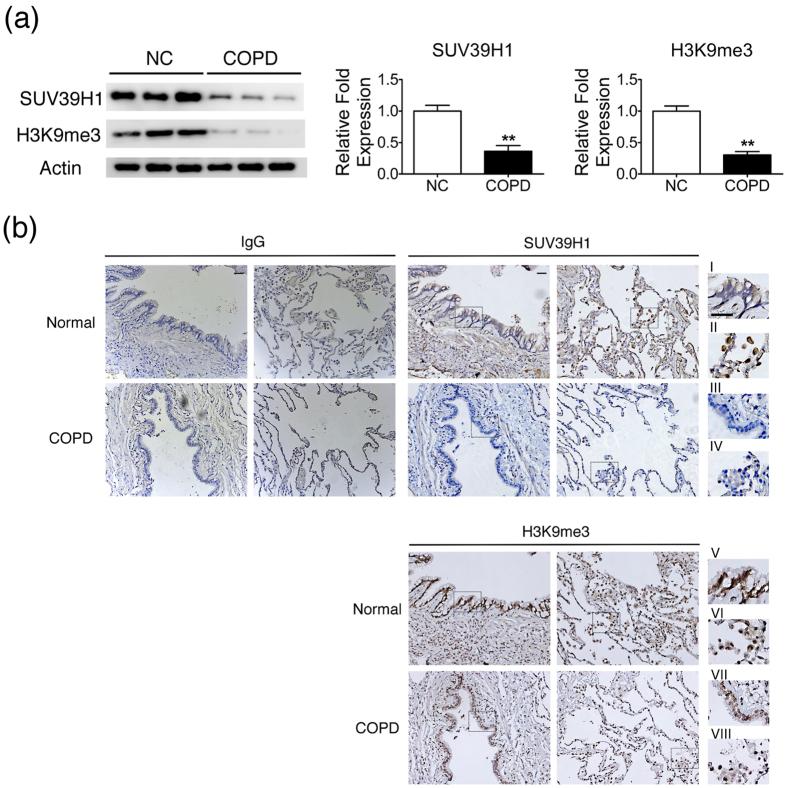
SUV39H1 and H3K9me3 levels are down-regulated in the lungs of COPD patients compared with control subjects. (**a**) Expression of SUV39H1 was reduced in human small airway epithelial cells derived from COPD patients(COPD HSAEpC) compared with normal HSAEpC(NC). The SUV39H1 protein levels were measured by western blot analysis.(**b**) SUV39H1 or H3K9me3 levels in lung tissues of normal or COPD subjects were estimated by immunohistochemistry. Immunohistochemical staining of enlarged regions of the lungs of patients with COPD showing reduced SUV39H1(i *vs* iii) or H3K9me3(v *vs* vii) levels in epithelial cells compared with normal subjects. Moreover, the SUV39H1(ii *vs* iv) or H3K9me3(vi *vs* viii) levels were reduced in macrophages or interstitial mononuclear cells. Normal mouse IgG was used as a negative control for both IHC staining. An enlarged view of the rectangular region was drawn with dashed lines. Scale bar, 200 μm. **p < 0.01.

We next asked whether airway epithelial cells also down regulate SUV39H1. To this end, we examined the level of SUV39H1 expression in primary human small airway epithelial cells(HSAEpCs) from both normal volunteers and patients with COPD(COPD HSAEpCs). Western blots of primary COPD HSAEpCs showed lower SUV39H1 or H3K9me3 expression than did normal HSAEpCs(Fig. 3a). To further confirm these observations, the SUV39H1 and H3K9me3 levels in the lung tissues of subjects without and with COPD were also estimated by immunohistochemistry. The staining of SUV39H1 and H3K9me3 in subjects with COPD was weaker than in those without COPD(Fig. 3b). This expression difference was seen in both airway epithelium(Fig. 3b-i,iii,v and vii) and macrophages(Fig. 3b-ii,iv,vi and viii). These results suggest that the SUV39H1 and H3K9me3 expression levels were reduced in circulating inflammatory cells(PBMCs) and in the lung, including airway epithelial cells and alveolar macrophages.

### The SUV39H1/H3K9me3 repressive mark at the *IL-8* promoter and IL-8 release in COPD HSAEpCs

**Figure 4:**
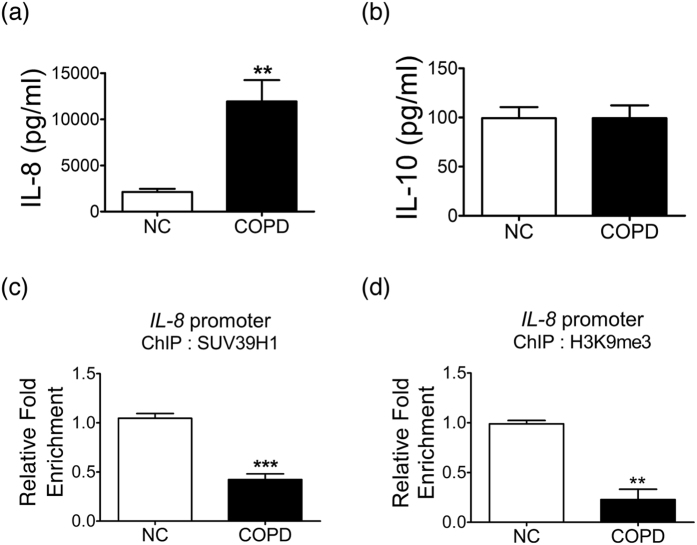
IL-8 is reduced in human small airway epithelial cells(HSAEpC) from COPD patients. (**a**) The IL-8 protein levels were reduced in human small airway epithelial cells derived from COPD(COPD HSAEpCs) patients compared with normal HSAEpCs(NC). The levels of IL-8 in supernatants were measured by ELISA.(**b**) However, the IL-10 levels in COPD HSAEpCs were similar to in normal control cells.(**c**) The recruitment of SUV39H1 in the *IL-8* gene promoter was reduced in COPD HSAEpCs compared with normal control. The enrichment of SUV39H1 was evaluated by chromatin immunoprecipitation(ChIP) analysis.(**d**) The enrichment of H3K9me3 in *IL-8* gene promoter was also reduced in COPD HSAEpCs. All data are presented as the mean ± SEM of at least three independent experiments. **p < 0.01 and ***p < 0.001.

Airway epithelial cells are also a source of inflammation. To explore the role of SUV39H1 in these cells, the release of inflammatory cytokines was examined. ELISA analysis revealed that IL-8 release was increased in COPD HSAEpCs compared with normal controls(Fig. 4a). As with PBMCs, no difference was observed in IL-10 release between normal or COPD HSAEpCs(Fig. 4b). We next analyzed the involvement of SUV39H1 in the predominant pro-inflammatory cytokine IL-8. Chromatin immunoprecipitation analysis showed that the recruitment of SUV39H1 to the *IL-8* gene promoter was reduced in COPD HSAEpCs compared with normal control(NC) samples(Fig. 4c). This reduction was accompanied with decreased levels of H3K9me3 at the promoter(Fig. 4d). Therefore, SUV39H1/H3K9me3 is reduced both at the global level and at the *IL-8* promoter, which is associated with the increased production of IL-8 in COPD HSAEpCs.

### Events of epigenetic control of transcription initiation induced by SUV39H1 inhibition at the native *IL-8* promoter

**Figure 5:**
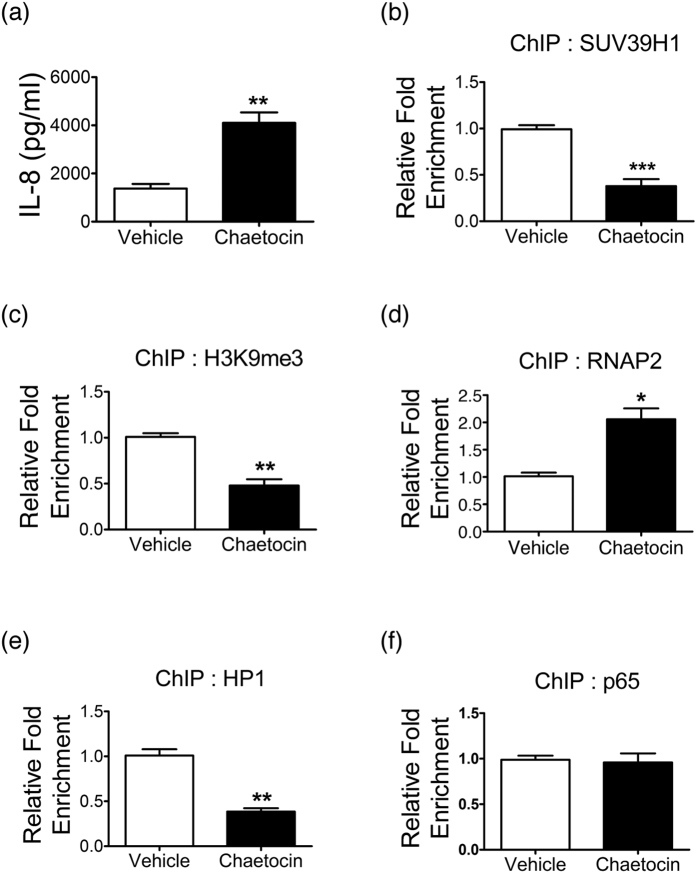
SUV39H1 inhibition is involved in the epigenetic modulation of the *IL-8* gene. (**a**) Normal human small airway epithelial cells(HSAEpCs) were treated with chaetocin(100 nM) for 16 hours, and IL-8 protein levels were evaluated by ELISA analysis.(**b**) The recruitment of SUV39H1 in the *IL-8* gene promoter was markedly reduced in HSAEpCs with SUV39H1 inhibitor(chaetocin) treatment compared with vehicle control(DMSO). The enrichment of SUV39H1 was evaluated by chromatin immunoprecipitation(ChIP) analysis.(**c**) The enrichment of H3K9me3 in the *IL-8* gene promoter was also decreased via the inhibition of SUV39H1 methyltransferase activity.(**d**) The IL8 levels were increased. Accordingly, the recruitment of RNA polymerase II(RNAP2) in *IL-8* gene promoter was robustly increased.(**e**) Similar to SUV39H1 modulation, the SUV39H1-interacting partner, HP1 was decreased in the epigenetic regulation complex with the blockade of SUV39H1 activity.(**f**) However, the enrichment of NF-κB subunit p65 was not significantly elevated in the *IL-8* gene promoter. All data are presented as the mean ± SEM of at least three independent experiments. *p < 0.05; **p < 0.01; and ***p < 0.001.

To elucidate the roles of SUV39H1 in the regulation of inflammation, we used HSAEpCs as cellular models. As for HSAEPCs, chaetocin treatment enhanced the production of IL-8(Fig. 5a). However, pro-inflammation cytokines were not significantly changed when the COPD HSAEPC cells were treated with SUV39H1 inhibitor(Supplementary Fig. S2B). These data may be resulted from very low levels of SUV39H1 in COPD HSAEPCs, compared with normal control. At the native *IL-8* promoter, the recruitment of SUV39H1 displayed a marked decrease following chaetocin treatment(Fig. 5b), which was accompanied by a reduction in the H3K9me3 level(Fig. 5c) and the associated HP1 protein(Fig. 5e). The decrease in the repressive mark was associated with a strong increase in the recruitment of RNA polymerase II(RNAP2)(Fig. 5d). NF-κB plays a central role in regulating the expression of inflammatory genes, including IL-8^25^. Our results showed that the occupancy of the NF-κB subunit p65 at the *IL-8* promoter did not significantly change(Fig. 5f). Collectively, these results demonstrate the involvement of SUV39H1 in the repressive chromatin state of the *IL-8* gene and that transcription initiation is controlled at a level downstream to NF-κB recruitment.

### The causative role of SUV39H1 in modulating the COPD pattern of inflammation in HSAEpCs

**Figure 6:**
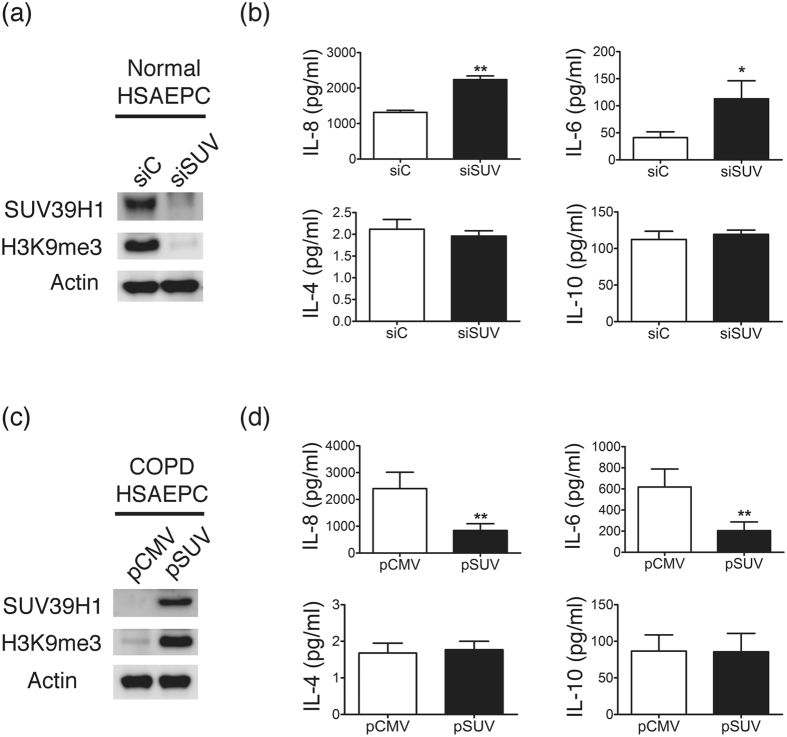
Effect of SUV39H1 knock down and overexpression on the inflammation response of human small airway epithelial cells. (**a**) Western blot analysis of SUV39H1 or H3K9me3 in normal small airway epithelial cells. HSAEpCs were transfected with SUV39H1 or control scramble siRNA. The SUV39H1 and H3K9me3 levels were reduced via siRNA transfection.(**b**) Cell supernatants were monitored for IL-8 secretion using an ELISA. The IL-8 and IL-6 levels were significantly increased in HSAEpCs knocked down with siSUV compared with siRNA control(siC). However, the IL-4 and IL-10 levels were not affected after transfection.(**c**) COPD HSAEpCs were transfected with SUV39H1(pSUV) or empty control vectors(pCMV). The SUV39H1 and H3K9me3 levels were increased via SUV39H1 expression vector transfection.(**d**) The IL-8 and IL-6 levels were significantly diminished in COPD HSAEpCs via SUV39H1 overexpression. There was no difference in the IL-4 and IL-10 levels between SUV39H1 overexpression and control cells. All data are presented as the mean ± SEM of at least three independent experiments. *p < 0.05 and **p < 0.01.

As chaetocin might not be sufficiently specific for SUV39H1, small interfering RNA–knockdown was used in normal HSAEpCs to confirm the functional role of SUV39H1. Western blot analysis revealed marked reduction of SUV39H1 protein by transfection with siSUV compared with scramble RNA(siC)(Fig. 6a). The inflammation response, including IL-8 and IL-6 cytokine secretion, was significantly increased by SUV39H1 knockdown(Fig. 6b). However, no difference was observed in the IL-4 or IL-10 levels. Therefore, both the pharmacological inhibition and knockdown of SUV39H1 recapture the COPD pattern of inflammation, thus suggesting a critical role of this protein in the disease.

To further confirm the causative role of reduced SUV39H1 in chronic inflammation in COPD, COPD HSAEpC cells were transfected with a SUV39H1 overexpression plasmid. SUV39H1 overexpression significantly rescued the level of H3K9me3(Fig. 6c). Notably, the inflammation response, including IL-8 and IL-6, but not IL-4 or IL-10, cytokines, was significantly decreased by SUV39H1 overexpression(Fig. 6d). These data suggest that the reduction of SUV39H1-H3K9me3 plays a vital role in inflammation in the COPD epithelium.

### SUV39H1/H3K9me3 expression and inflammation in a murine smoking model

**Figure 7:**
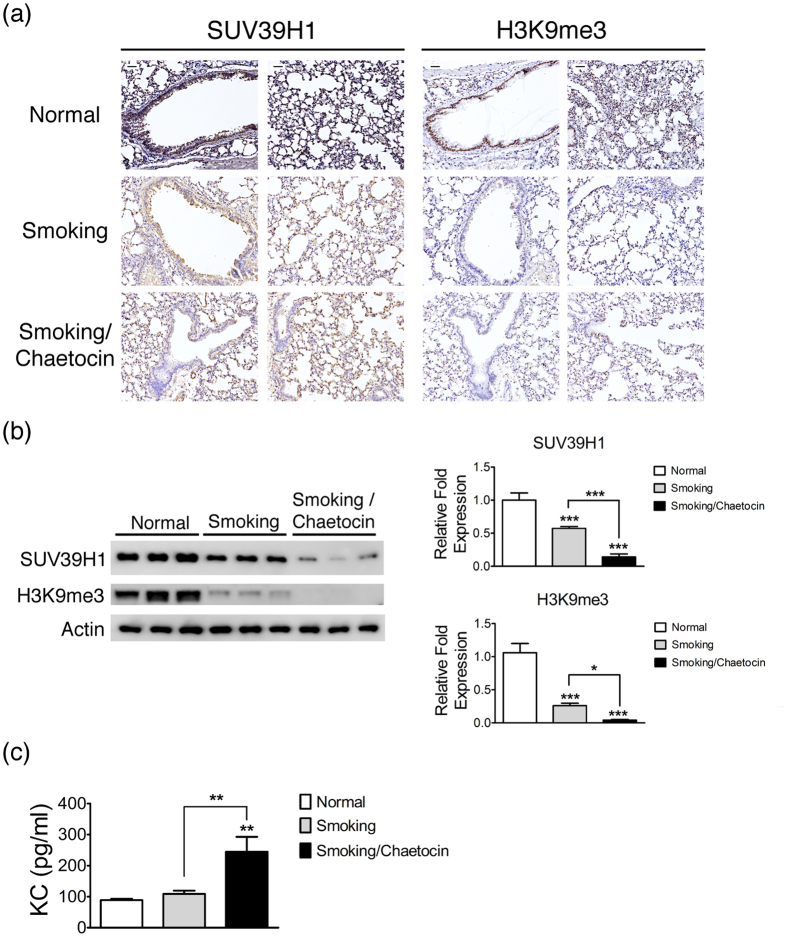
SUV39H1 repression increases the inflammation response in cigarette smoke exposure mice. (**a**) SUV39H1 or H3k9me3 levels were visualized by immunohistochemical staining in the lungs of cigarette smoke filtered control(normal), cigarette smoke exposure(smoking), or smoking combined with chaetocin administration(smoking/chaetocin) mice. Smoking and smoking/chaetocin mice displayed slightly and markedly reduced SUV39H1 expression in the epithelium compared with control mice, respectively. The H3K9me3 levels were also considerably decreased in smoking and smoking/chaetocin mice. Scale bar, 200 μm.(**b**) Western blot analysis of SUV39H1 or H3k9me3 showed a similar trend as the immunohistochemical staining results. The H3k9me3 levels were significantly reduced in smoking and smoking/chaetocin mice compared with control mice.(**c**) Chaetocin treatment accelerates the protein levels of a murine IL-8 homologue, KC, in the lung tissue induced by smoking. The KC expression of the smoking(gray bars) or smoking and chaetocin(black bars) treated mice(n = 6 animals per group) is depicted compared with control mice. *p < 0.05; **p < 0.01; and ***p < 0.001.

To demonstrate that patients with COPD will develop a down-regulation of SUV39H1, which will further complicate the inflammation, we established a murine smoking model of COPD. Mice that were subjected to cigarette smoke exposure developed pulmonary inflammation and emphysema over a 12-week time course(Supplementary Fig. S3). Figure 7a shows data for the modulation of the SUV39H1 and H3k9me3 levels in murine lungs in this experimental model. Immunohistochemical staining of the lung in smoking mice displayed reduced SUV39H1 expression in the epithelium compared with control mice. Chaetocin injection of smoking mice further decreased the SUV39H1 expression. The H3K9me3 expression levels were also considerably decreased in smoking and smoking/chaetocin mice, with a similar trend. Accordingly, immunoblotting confirmed that the SUV39H1 or H3k9me3 protein levels were significantly decreased in smoking mice compared with control mice and that there was a further decrease in smoking/chaetocin mice(Fig. 7b). As with the human data, smoking COPD mice had higher level of KC, the murine homologue of IL-8, whereas chaetocin treatment further enhanced the levels(Fig. 7c). Although chaetocin was initially found to inhibit the SUV39H1 activity, recent reports have shown that chaetocin reduced SUV39H1 expression by inhibiting Hsp90-mediated SUV39H1 stability^26^. Similarly, the SUV39H1 levels were markedly reduced in our chaetocin-treated mice. HSAEpCs showed a similar trend for COPD. SUV39H1 knockdown by RNAi decreased the H3K9me3 levels and enhanced the production of cytokines with the same pattern observed for chaetocin. Overall, these data implicate a contributing role for SUV39H1 repression in COPD.

## Discussion

The aberrant regulation of inflammation at the epigenetic level has been critically implicated in abnormal inflammation in COPD. In addition to epigenetic regulation via reduced HDAC2, the present study extends our understanding to the field of histone methylation. We have shown that the global levels of SUV39H1 and H3K9me3 are reduced in PBMC, HSAEpCs and lung tissues from patients with severe COPD. The reduced SUV39H1 in PBMCs was associated with the serum levels of IL-6, IL-8 and poor lung function. *In vitro* studies clearly demonstrated that SUV39H1 controlled a panel of inflammatory genes similar to those augmented- and sustained-released from COPD PBMCs. SUV39H1 overexpression further indicated that targeting upstream of SUV39H1 or its regulatory mechanisms might be a potential therapy for COPD.

The present study establishes, for the first time, a link between the modulation of SUV39H1 and COPD. The SUV39H1 levels were reduced in PBMCs, which were correlated with systemic inflammation and poor lung function. On examination of the lung tissue from patients with severe COPD, our IHC studies revealed weaker expression of both proteins in bronchial epithelial cells and lung inflammatory cells, mainly in the alveolar macrophages. Western blot analysis of HSAEpCs from different donors confirmed this phenomenon in this cell type. Therefore, the reduction in SUV39H1/H3K9me3 generally involves circulating and lung inflammatory cells as well as airway epithelial cells. Animal studies indicate that smoking induces the same reduction when emphysema develops. However, SUV39H1 does not decrease in healthy smokers and only has a trend of decreasing in GOLD 1–2 patients. It is likely that reduction of SUV39H1 is established in the late stages of the disease. Alternatively, a subset of patients might be prone to this defect in response to noxious gas and may thus progress rapidly to severe disease. Indeed, in the ECLIPSE cohort, only a proportion of the patients showed lung function decline^27^. Similarly, only 28% of the patients had high levels of two or more inflammatory biomarkers in serum, whereas 16% demonstrated persistent inflammation^11^. Whether SUV39H1 is implicated in these patients merits further study.

The mechanism whereby SUV39H1 is reduced remains unknown. As mutations or polymorphisms of the *SUV39H1* gene have not been reported in genetics studies for COPD^28^, mechanisms involving transcriptional or post-transcriptional regulation and occurring in general might be implicated in disease progression. Indeed, we did not detect differences in the levels of *SUV39H1* mRNA between normal and COPD PBMCs(data not shown). A post-transcriptional mechanism seems to be the major defect. The stability and activity of SUV39H1 might be regulated via various pathways^29^,^30^,^31^,^32^,^33^,^34^,^35^. SIRT1, as an oxidative stress sensor, controls the global levels of SUV39H1 by increasing its stability^29^. Because SIRT1 is reduced in COPD^15^, SUV39H1 might be down-regulated through a proteolysis mechanism. This hypothesis might also explain the slight increase in SUV39H1 in healthy smokers who had intact stress responses to smoking.

H3K9 methylation regulates a subset of LPS-inducible inflammatory genes^20^. Vascular smooth muscle cells derived from diabetic mice exhibit a persistent inflammatory phenotype with increased release of IL-6, MCSF, and MCP-1. Decreased H3K9me3 and SUV39H1 occupancy at the promoters of those genes is causatively implicated in this inflammatory phenotype^23^. Moreover, the SUV39H1–H3K9me3–HP1α pathway participates in silencing TH1, but not TH2, loci^22^. Consistent with these observations, we found this repressive mechanism is implicated in the regulation of a panel of pro-inflammatory genes instead of TH2 genes. Noteworthy, the products of such pro-inflammatory genes have long been implicated in COPD^36^. Therefore, a loss of the repressive H3K9me3 due to decreased SUV39H1 plays a crucial role in the sustained and augmented inflammation that is associated with COPD. The reduction in SUV39H1 modulation might enhance cytokine secretion into the inflammatory environment in the COPD airways and circulation, which could amplify both the inflammation response and disease progression. The defect in SUV39H1/H3K9me3 is implicated in the abnormal inflammation of COPD.

In the ECLIPSE study, the systemic inflammatory network pattern associated with COPD included WBC, CRP, IL-6 and fibrinogen^27^. Although the levels of IL-8 and TNFα were higher in COPD, they were also elevated in healthy smokers. In the present study, we showed a significant negative correlation of the serum IL-6 level with SUV39H1. However, the significance for IL-8 was only found after excluding the healthy smokers from the analysis. In addition, whereas IL-8 was induced by higher H_2_O_2_ concentrations(300 μM) in normal PBMCs, IL-6 did not respond to all of the H_2_O_2_ concentrations(0–300 μM) used in this study. Therefore, our results are in agreement with the ECLIPSE study. Although both IL-6 and IL-8 are SUV39H1-controlled genes, their mechanism levels regulate the IL-8 expression.

**Figure 8:**
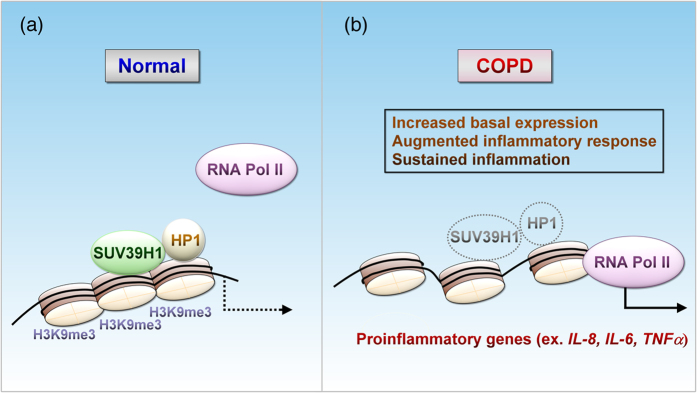
A scheme of SUV39H1 down-regulation involved in the dysregulation of inflammation in COPD. Reduction of SUV39H1 modulation releases repressive chromatins and enhances cytokine secretion to the inflammatory environment in COPD, which potentially amplifies the inflammation response and disease progression.(**a**) In normal subjects, SUV39H1 mediates the repressive chromatin state of pro-inflammation genes, e.g., IL-8, IL-6, and TNFα, but not the one encoding Th2 cytokines, through an interplay with H3K9me3 and HP1. By keeping these genes in check, cells are stable and the inflammatory response is tightly controlled.(**b**) In patients with COPD, a profound reduction in SUV39H1 releases the repression of such genes and resets the production of cytokines to a higher level in the chronic inflammatory milieu. Upon further stimulation(ex. oxidative stress), transactivation of these genes results in prolonged enhancement in an unchecked manner.

Oxidative stress plays an important role in the pathogenesis of COPD^37^. We consistently showed an enhanced response of COPD PBMCs to H_2_O_2_ in terms of IL-8 induction^24^. The reduced NKRF expression conferred this enhancement. With the same amount of NF-κB bound to the *IL-8* promoter, the release of NKRF repression can further recruit RNAP2 and thereby stimulate transcription. However, only some NKRF-control genes have been reported. Whether NKRF abnormality leads to all inflammation in COPD remains questionable. In contrast, SUV39H1 holds genes across pro-inflammation and keeps the TH1 reactions in check, which seems to be a more universal mechanism. Without the further recruitment of NF-κB, inhibition of SUV39H1 by chaetocin can induce IL-8 transcription by erasing the H3K9me3 repressive marking and removing the HP-1 co-repressor(Fig. 8). Therefore, SUV39H1/H3K9me3 adds an additional regulatory level to gene transactivation^20^. At lower levels of the repressive mark, SUV39H1-controlled genes will increase their basal expression and have enhanced and sustained response to stimulations, such as excessive oxidative stress in COPD.

SUV39H1 controls a distinct panel of pro-inflammatory genes that are implicated in COPD chronic inflammation. A loss of the SUV39H1/H3K9me3 repressive mechanism amplifies and prolongs the inflammatory response in COPD through an epigenetic mechanism that is downstream of NF-κB binding to the promoter. Our results also suggest that the maintenance of SUV39H1 expression may be a potential new treatment for COPD.

## Methods

### Study population

Peripheral blood mononuclear cells(PBMCs) were obtained form 10 healthy non-smokers, 10 healthy smokers and 25 patients with stable COPD. Details on the COPD patients are provided in Supplementary Table 1. Patients with COPD were diagnosed, and their severity was graded according to the guidelines of the Global Initiative for Obstructive Lung Disease^1^. Patients were in a stable condition without acute exacerbations within 3 months, defined as no requirement for antibiotic or oral corticosteroid therapy and no change in respiratory symptoms. Patients were excluded if they had known asthma; bronchiectasis; tuberculosis; pneumoconiosis; heart disease; malignancy; or other infectious, inflammatory or metabolic conditions that clearly induced symptoms of chest tightness and dyspnea. Normal subjects were defined as having FEV1 values of greater than 80% of the predicted values and FEV1/FVC ratios greater than 75% without any known medical diseases, and they had not experienced any acute infections, including upper airway infection, in 3 months. Patients were followed for at least one year to determine the rate of acute exacerbation, FEV1 decline rate and other clinical outcomes, including events of newly developed co-morbidities, e.g., cardiovascular events, cancer, and mortality. Full informed consent was obtained from all participants or their parents before sample collection. The study protocol was approved by Taipei Medical University-Joint Institutional Review Board(TMU-JIRB, no. 201502024). All experiments were performed in accordance with the relevant guidelines and regulations.

### Cell Culture and Stimulation

PBMCs were isolated from whole blood by Ficoll-Hypaque density gradient centrifugation as previously described by our group^24^. PBMCs were maintained in continuous cell culture at 37 °C and 5% CO_2_ in RPMI 1640 medium containing 10% fetal bovine serum(FBS) and 2 mM L-glutamine. Normal bronchial cells(HSAEpCs) and diseased COPD airway cells(Lonza, Basel, Switzerland) were grown in commercialized medium according to the instructions. In inflammation stimulation experiments, PBMCs or HSAEpCs were incubated with H_2_O_2_(100–300 μM), IL-1β(10 ng/ml), and chaetocin(10 μM) overnight or for the indicated duration. Supernatants were collected and the levels of pro-inflammatory cytokines were measured by ELISA.

### Western Blot

Cellular proteins were subjected to 10–12% SDS-polyacrylamide gel electrophoresis and blotted onto polyvinylidene difluoride membrane. Western blot was performed as previously described^24^. We employed antibodies against SUV39H1(Cell Signalling, Hitchin, UK), H3K9me3(Novus Biologicals, Littleton, CO, USA), and β-actin(Abcam, Cambridge, UK). The intensities of immunoreactive bands were quantified using Image Gauge software(Fuji Film, Tokyo, Japan).

### Enzyme-Linked Immunosorbent Assay(ELISA)

The TNF-α, IL-4, IL-6, IL-8, and IL-10 levels in the supernatants were measured by commercial ELISA kits(eBioscience, San Diego, CA, USA) according to the manufacturer’s instructions.

### Transfection and RNA interference(RNAi)

HSAEpCs were transiently transfected using the Nucleofector system from Amaxa Biosystems. After centrifugation, 5 × 10^6^ cells were suspended in 100 μl of pre-warmed Nucleofector solution containing a 500-nM final concentration of small interfering RNAs(siRNAs) targeting SUV39H1(Dharmacon, Lafayette, CO, USA) or the full-length expression vector for SUV39H1(OriGene Technologies, Rockville, MD, USA) using the manufacturer’s protocols. The concentrations of plasmids and siRNAs were optimized. The samples were transferred into an electroporation cuvette, and transfections were performed with the manufacturer’s programs. After nucleofection, the cells were immediately transferred into pre-warmed complete RPMI medium and cultured at 37 °C in a humidified atmosphere of 5% CO_2_.

### Immunofluorescence and Immunohistochemical(IHC) analysis

Aliquots of 1 × 10^5^ cells from PBMCs were cytospun for 5 minutes at 500 rpm using a centrifuge(Thermo Shandon, Pittsburgh, PA, USA). The slides were air dried and fixed in methanol for 10 minutes. Then, the cells were blocked with 1% BSA/PBS at room temperature for 30 min and incubated with antibodies specific for H3K9me3(GeneTex, San Antonio, TX, USA) or SUV39H1(Novus Biologicals, Littleton, CO, USA) at room temperature for 40 min. After washing, the cells were incubated with an anti-rabbit antibody Alexa Fluor^®^ 488(Abcam, Carlsbad, CA, USA) at room temperature for 1 hr. After washing, the cells were incubated with 4′,6-diamidino-2-phenylindole dihydrochloride(DAPI) at room temperature for 10 min. Immunostaining cells were mounted on slides with anti-fade mounting medium(DAKO, Japan). Lung tissues were obtained from COPD patients(patients undergoing lung surgery for peripheral lung tumor removal). Lung function testing was performed before lung cancer surgery. Normal control tissues were derived from noninvolved lung segments of the tumor lesion. IHC was performed on paraffin-embedded human or mouse lung tissue sections using rabbit antibodies against SUV39H1 and H3K9me3, as previously described^29^. Images were acquired at 200x magnification and analyzed by Nikon NIS Elements D Imaging Software Analysis(Nikon, Tokyo, Japan).

### Smoking mouse model of COPD

Six-week-old male BALB/c mice(n = 6 animals/group) were obtained from National Laboratory Animal Center(Taipei, Taiwan). The animals were housed in plastic cages and were provided Lab Diet 5001(PMI Nutrition International,. Brentwood, MO, USA) and water ad libitum during acclimatization. BALB/c mice were exposed to cigarette smoke using a cigarette smoke chamber equipped with whole-body exposure cages. The main-stream smoke from the combustion of 12 reference cigarettes(3R4F; Tobacco and Health Research Institute, KY, USA) were introduced into the whole-body exposure cages for a period of approximately 50 minutes/day, 5 days/week, for 12 weeks. Control mice were exposed to cigarette smoke-free HEPA-filtered air. The mass and number concentrations were monitoring using DusTrak(TSI DRX 8534) and P-Trak(TSI 8525) instruments, respectively. For the smoking/chaetocin group, smoking mice were given chaetocin(10 mg/kg body weight; Sigma-Aldrich Co. LLC.) by intraperitoneal injection on the final day. All animals were sacrificed at the end point after smoking challenge. The animal experiments were performed in compliance with the animal and ethics review committee of the Laboratory Animal Center at the Taipei Medical University, Taiwan(LAC-2014-0267).

### Chromatin Immunoprecipitation(ChIP) Assay

ChIP assays were performed as previously described^24^. Briefly, protein-DNA complexes were cross-linked by 1% formaldehyde for 10 min at 37 °C. Cells were resuspended in 200 μl of SDS lysis buffer and subjected to three cycles of sonication on ice with 10-S pulses. Then, sonicated samples were centrifuged to spin down cell debris, and the soluble chromatin solutions were immunoprecipitated using antibodies specific for p65(Bethyl Lab., Montgomery, TX, USA), HP1(GeneTex, San Antonio, TX, USA), SUV39H1, H3K9me3, and RNA polymerase II(Millipore, Billerica, MA, USA). The final immunoprecipitated DNA(IP DNA) were resolved in nuclease-free H_2_O and the DNA solutions were quantified by quantitative PCR. Primers amplifying irrelevant segments of DNA in the IL-8 gene, the 3′ UTR sites, were used as site-specific binding controls.

### Statistical Analysis

Data were analyzed with GraphPad Prism 5.0 software(GraphPad Software, San Diego, CA, USA). Unless stated otherwise, all data are presented as the mean ± SEM and are representative of at least three independent experiments. Comparisons between two groups were analyzed using the two-tailed unpaired t tests when the data were normally distributed; otherwise, Mann-Whitney analysis was performed. Differences were considered significant at p < 0.05.

## Additional Information

**How to cite this article**: Chen, T.-T. *et al*. SUV39H1 Reduction Is Implicated in Abnormal Inflammation in COPD. *Sci. Rep.* **7**, 46667; doi: 10.1038/srep46667(2017).

**Publisher's note:** Springer Nature remains neutral with regard to jurisdictional claims in published maps and institutional affiliations.

## Supplementary Material

Supplementary Data
