# Corrigendum: SUV39H1 Reduction Is Implicated in Abnormal Inflammation in COPD

**DOI:** 10.1038/srep46924

**Published:** 2017-12-22

**Authors:** Tzu-Tao Chen, Sheng-Ming Wu, Shu-Chuan Ho, Hsiao-Chi Chuang, Chien-Ying Liu, Yao-Fei Chan, Lu-Wei Kuo, Po-Hao Feng, Wen-Te Liu, Kuan-Yuan Chen, Ta-Chih Hsiao, Jer-Nan Juang, Kang-Yun Lee

Scientific Reports 7: Article number: 46667; 10.1038/srep46667 published online: 04 20 2017; updated: 12 22 2017.

The original version of this Article contained errors in the numbering of affiliations where Affiliations 1 and 2 were reversed.

In addition, the title and legend of Figure 4 contained errors.

“**Figure 4: IL-8 is reduced in human small airway epithelial cells(HSAEpC) from COPD patients**. (**a**) The IL-8 protein levels were reduced in human small airway epithelial cells derived from COPD(COPD HSAEpCs) patients compared with normal HSAEpCs(NC). The levels of IL-8 in supernatants were measured by ELISA. (**b**) However, the IL-10 levels in COPD HSAEpCs were similar to in normal control cells. (**c**) The recruitment of SUV39H1 in the *IL-8* gene promoter was reduced in COPD HSAEpCs compared with normal control. The enrichment of SUV39H1 was evaluated by chromatin immunoprecipitation(ChIP) analysis. (**d**) The enrichment of H3K9me3 in *IL-8* gene promoter was also reduced in COPD HSAEpCs. All data are presented as the mean ± SEM of at least three independent experiments. **p < 0.01 and ***p < 0.001”.

now reads:

“**Figure 4: IL-8 is induced in human small airway epithelial cells(HSAEpC) from COPD patients**. (**a**) The IL-8 protein levels were induced in human small airway epithelial cells derived from COPD(COPD HSAEpCs) patients compared with normal HSAEpCs(NC). The levels of IL-8 in supernatants were measured by ELISA. (**b**) However, the IL-10 levels in COPD HSAEpCs were similar to in normal control cells. (**c**) The recruitment of SUV39H1 in the *IL-8* gene promoter was reduced in COPD HSAEpCs compared with normal control. The enrichment of SUV39H1 was evaluated by chromatin immunoprecipitation(ChIP) analysis. (**d**) The enrichment of H3K9me3 in *IL-8* gene promoter was also reduced in COPD HSAEpCs. All data are presented as the mean ± SEM of at least three independent experiments. **p < 0.01 and ***p < 0.001”.

These errors have now been corrected in the HTML and PDF versions of the Article.

