# Supplementary material for: SUV39H1 Reduction Is Implicated in Abnormal Inflammation in COPD
Source: Sci Rep. 2017 Apr 20;7:46667. doi: 10.1038/srep46667 (PMC5397975; doi:10.1038/srep46667)
Supplement: Supplementary Data [file srep46667-s1.pdf]

SUV39H1 Reduction Is Implicated in Abnormal Inflammation in COPD

Tzu-Tao Chen[1,2], Sheng-Ming Wu[1], Shu-Chuan Ho[1,3], Hsiao-Chi Chuang[1,3,4], Chien-Ying Liu[5], Yao-Fei Chang[1], Lu-Wei Kuo[1], Po-Hao Feng[1,4], Wen-Te Liu[1,3,4,6], Kuan-Yuan Chen[1,2], Ta-Chih Hsiao[7], Jer-Nan Juang[6], Kang-Yun Lee[1,2,3]

[1]Division of Pulmonary Medicine, Department of Internal Medicine, Shuang Ho Hospital, Taipei Medical University, New Taipei City, Taiwan

[2]Graduate Institute of Clinical Medicine, College of Medicine, Taipei Medical University, Taipei, Taiwan

[3]School of Respiratory Therapy, College of Medicine, Taipei Medical University, Taipei, Taiwan

[4]Division of Pulmonary Medicine, Department of Internal Medicine, School of Medicine, College of Medicine, Taipei Medical University, Taipei, Taiwan

[5]Division of Pulmonary Oncology and Interventional Bronchoscopy, Department of Thoracic Medicine, Linkou Chang Gung Memorial Hospital, Taoyuan, Taiwan

[6]Department of Engineering Science, National Cheng Kung University, Tainan, Taiwan

[7]Graduate Institute of Environmental Engineering, National Central University, Taoyuan, Taiwan

Supplementary Table 1. Characteristics of the study subjects with COPD

Characteristic	Normal		COPD	
GOLD stage	Non-smoking	Smoking	I or II	III or IV
Subjects	10	10	12	13
Age years	69.60 ± 0.91	68.67 ± 0.97	67.67 ± 1.96	65.62 ± 1.97
Sex M/F	4/6	10/0	12 / 0	12 / 1
Smoking Yes/No	0/10	10/0	12 / 0	10 / 3
FEV1/FVC %	100.30 ± 5.28	100.83 ± 4.25	66.65 ± 3.07	52.08 ± 2.70
FEV1 % pred	100.26 ± 1.86	100.78 ± 2.22	67.61 ± 4.15	35.54 ± 2.96
BMI kg/m^2	25.52 ± 1.22	26.21 ± 2.01	21.79 ± 1.22	22.85 ± 1.39
Medications				
Corticosteroids			0	2
ICS (β2-agonist+Steroids)			3	6
Xanthine			3	7

Data are expressed as n or mean±SEM. COPD: chronic obstructive pulmonary disease; GOLD: Global Initiative for Chronic Obstructive Lung Disease; M: male; F: female; FEV1: forced expiratory volume in 1 s; FVC: forced vital capacity; % pred: % predicted; BMI: body mass index; ICS: inhaled corticosteroid.

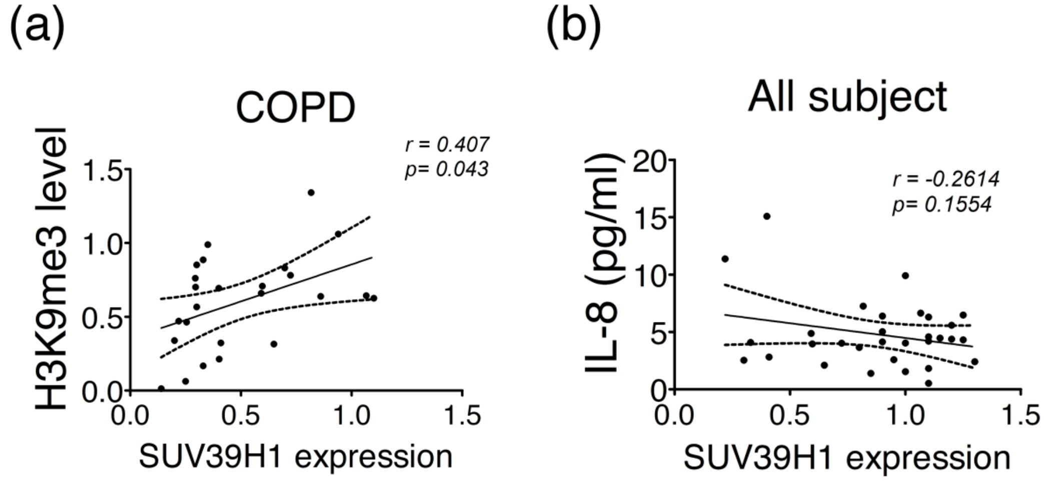

Supplementary figure S1. Correlation between H3K9me3 or IL-8 and SUV39H1 in COPD or all subjects. (A) A significant correlation was observed between the H3K9me3 and SUV39H1 protein levels of PBMC samples in COPD subjects. (B) A trend of negative correlation was observed between IL-8 and SUV39H1 levels of serum and PBMC samples in all subjects, respectively.

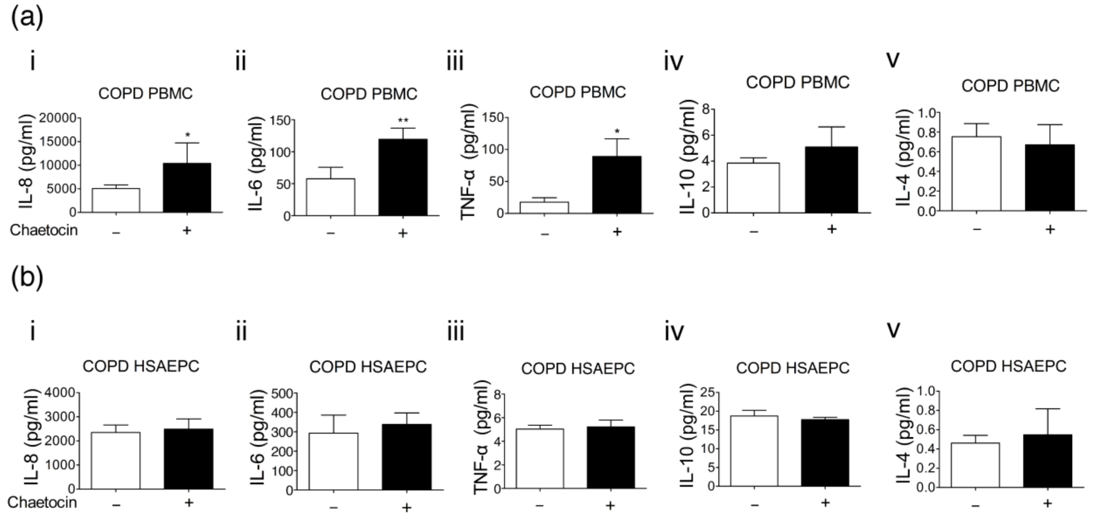

Supplementary figure S2. SUV39H1 inhibition augments a pro-inflammatory response in COPD PBMCs.

(A) COPD PBMCs were seeded in the presence of SUV39H1 inhibitor, chaetocin, at 100 nM. These inflammatory cytokines in supernatant were measured by ELISA before overnight stimulation. A panel of inflammatory cytokines including IL-8, IL-6, and TNF-α, but not IL-10 or IL-4 was induced by chaetocin in COPD PBMCs. (B) However, pro-inflammatory cytokines were not significantly changed when the COPD HSAEPC cells were treated with SUV39H1 inhibitor

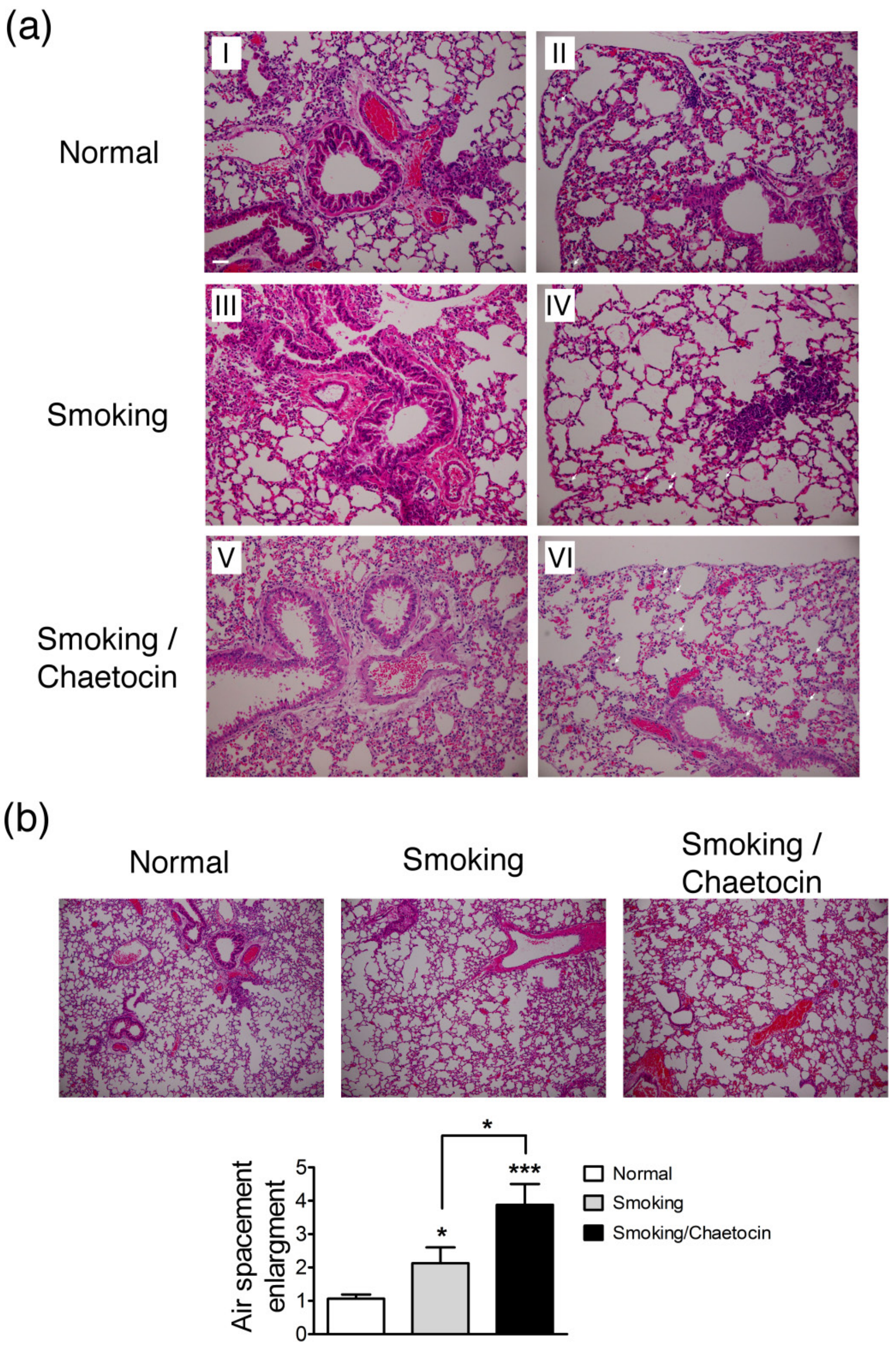

Supplementary figure S3. Cigarette smoking contributes to the development of pulmonary inflammation and emphysema. (A) Hematoxylin and eosin (H&E) staining of lung sections in normal, smoking and smoking/chaetocin mice, showing macrophages or interstitial mononuclear cells (arrow) over a 12-week time course of smoking exposure. Scale bars, 200 µm. (B) H&E-stained lung sections of normal,

smoking and smoking/chaetocin mice (upper panel) and quantitative analysis of alveolar airspace enlargement (lower panel). Average airspace area was calculated by dividing the total airspace by the total number of alveoli in three randomly selected fields at × 100 magnification in each section. Values shown are relative airspace area, with the value of normal mice arbitrarily set to 1.
